# Subcapsular renal hematoma after ureterorenoscopy

**DOI:** 10.1002/iju5.12464

**Published:** 2022-05-13

**Authors:** Narumi Harada, Junji Yatsuda, Ryoma Kurahashi, Yumi Fukushima, Takuya Segawa, Takanobu Motoshima, Yoji Murakami, Takahiro Yamaguchi, Yutaka Sugiyama, Tomomi Kamba

**Affiliations:** ^1^ Department of Urology Kumamoto University Kumamoto Japan

**Keywords:** hematoma, infections, kidney, lithotripsy, ureterorenoscopy

## Abstract

**Introduction:**

Subcapsular renal hematoma after ureterorenoscopy using a holmium yttrium‐aluminum‐garnet laser is a rare complication. We experienced a case of subcapsular hematoma after ureterorenoscopy.

**Case presentation:**

The patient was a 56‐year‐old man with a history of hypertension and coronary vasospastic angina, and he was taking antiplatelet drugs. He had the middle and lower calyx stones measured 36 mm in diameter of the right kidney. We performed ureterorenoscopy, which was completed about 2 h without intraoperative complications. We could not remove the stone completely. After the surgery, the patient developed a fever and complained of right back pain. Computed tomography showed several residual stones formed a stone street, obstructing the stent and resulting in grade 3 hydronephrosis. Furthermore, the right subcapsular renal hematoma infection had detected. Percutaneous hematoma drainage and percutaneous nephrostomy were performed.

**Conclusion:**

Subcapsular renal hematoma after ureterorenoscopy is an uncommon complication but should be kept in mind.

**Abbreviations & Acronyms**
CRP: C‐reactive protein
CT: computed tomography
ECIRS: endoscopic combined intrarenal surgery
ESWL: extracorporeal shock wave lithotripsy
Ho‐YAG: holmium yttrium‐aluminum‐garnet
POD: postoperative day(s)
URS: ureterorenoscopy

**Keynote message**

Subcapsular renal hematoma after URS using a Ho‐YAG laser is a rare complication. If patients develop a fever or complain of back pain after URS, subcapsular hematoma should be considered.

## Introduction

Currently, URS is the standard treatment for ureteral stones and renal stones measuring ≤2 cm. Transurethral surgery is associated with few serious complications and is reliable regarding crushing stones. Therefore, URS is performed in many institutions as a routine clinical practice in urology. We experienced a case of subcapsular hematoma after URS, which is a known serious complication after ESWL^1^ but a very rare complication after URS.

## Case presentation

**Fig. 1:**
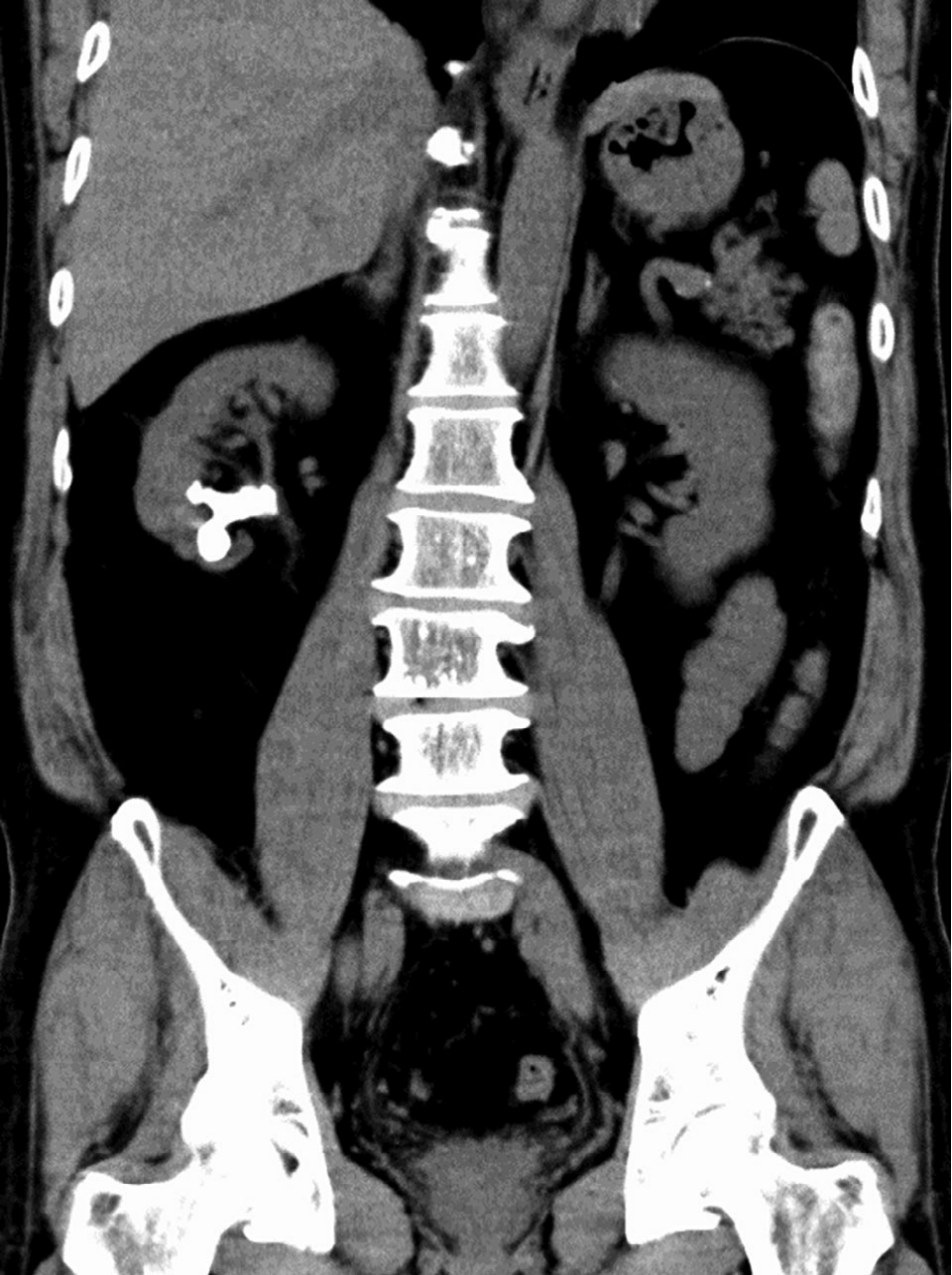
CT showing right middle and lower calyx stones (the longest diameter: 36 mm, the average CT value: 798 Hounsfield unit).

A 56‐year‐old male patient was admitted to our hospital because of a right renal stone, with no stone‐related symptoms. CT revealed the middle and lower calyx stones, without hydronephrosis (Fig. 1). He had a history of treatment for renal stone and had been in a stone‐free status for a while.

**Fig. 2:**
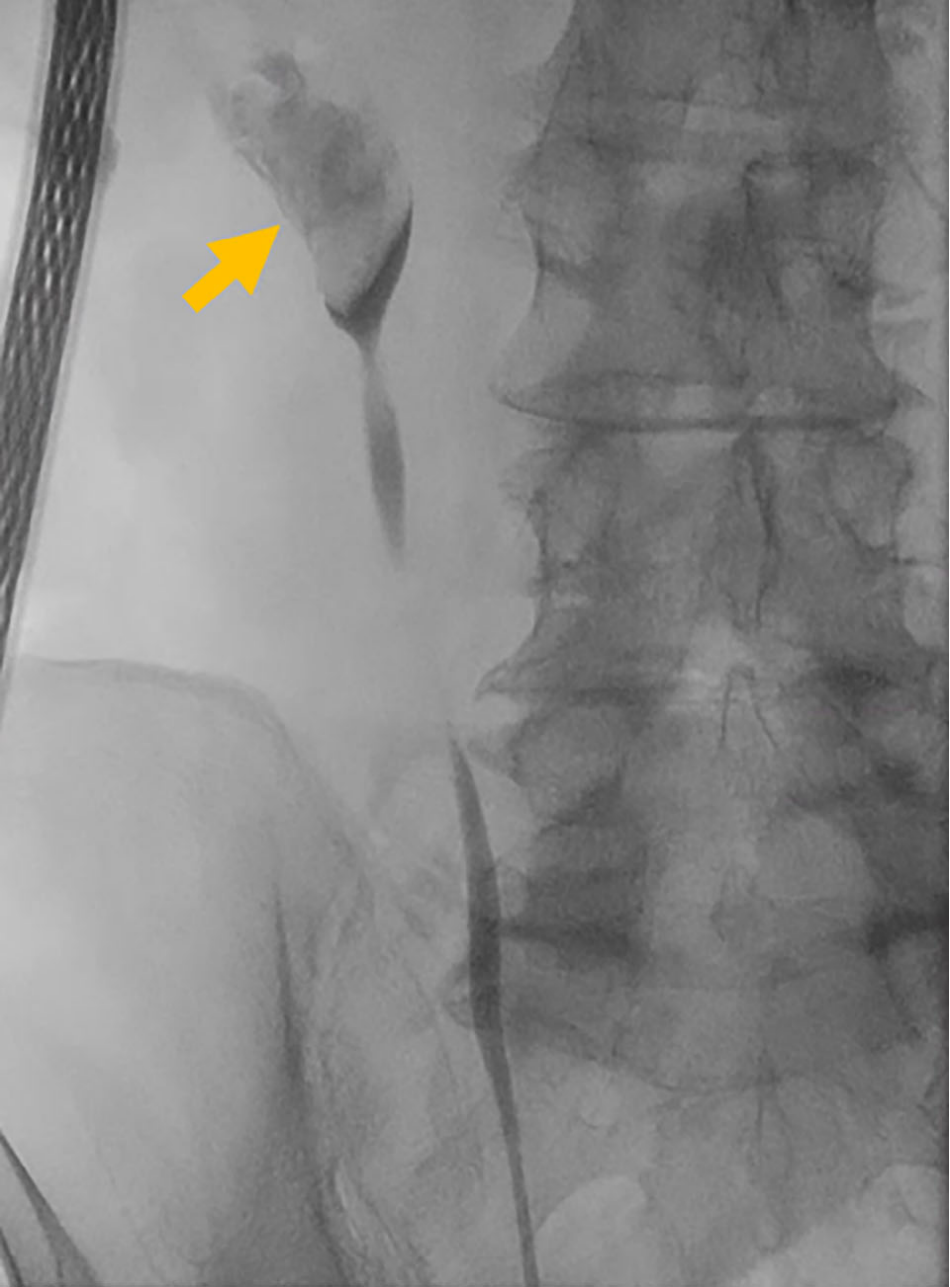
Retrograde pyelography showing that the stone further grew into the renal pelvis (arrow). [Colour figure can be viewed at wileyonlinelibrary.com]

Because he had developed coronary spasmodic angina as a postoperative complication of the previous percutaneous lithotripsy, we decided to perform URS without cessation of antiplatelets in multiple sessions, if necessary. The stone was found to grow further and extend into the renal pelvis (Fig. 2). The renal pelvic stone was crushed with a Ho‐YAG laser under an 8.4‐Fr. flexible ureteroscopy through a 12/14‐Fr. ureteral access sheath. We retrieved some fragments, but the others remained in‐situ because of the limitation of operation time. A 6‐Fr. 26‐cm double pig‐tail ureteral stent was placed in the right ureter. The operation time was 2 h and 8 min.

**Fig. 3:**
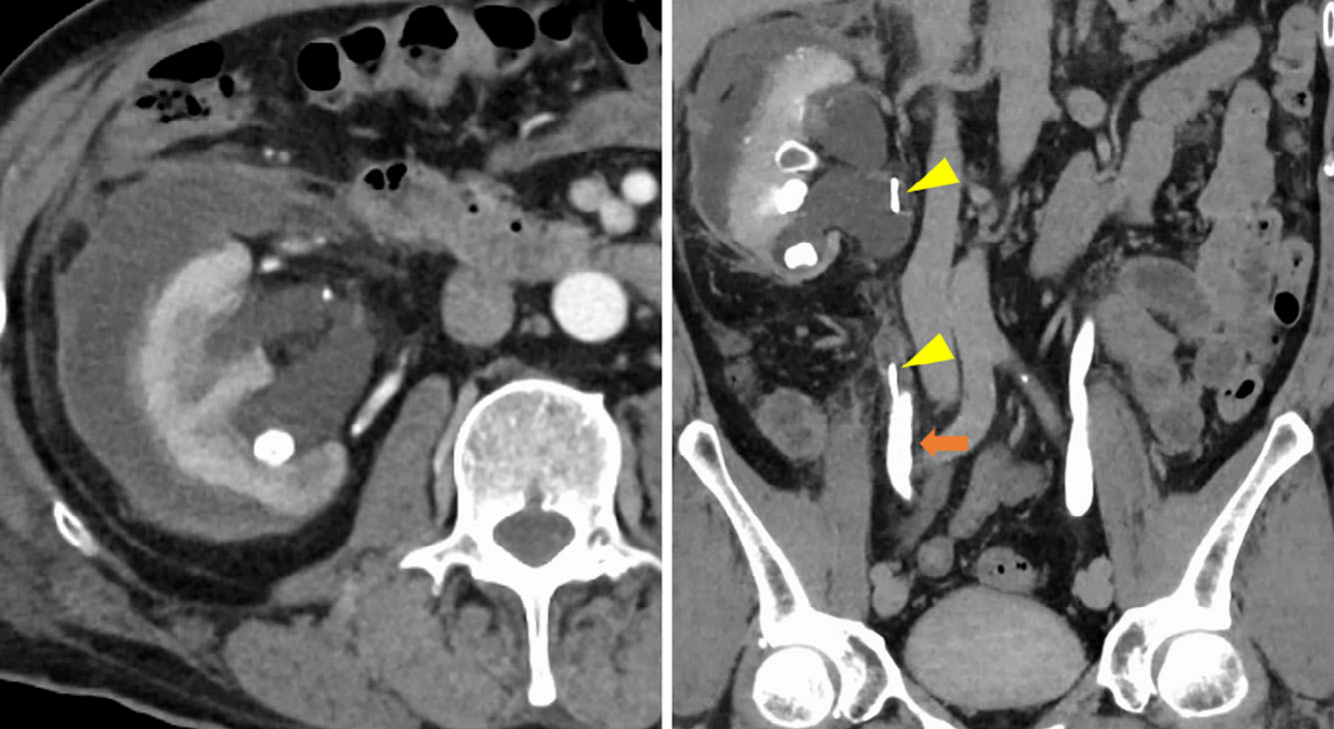
CT showing right hydronephrosis and a subcapsular renal hematoma (arrow: stone street, arrowhead: ureteral stent). [Colour figure can be viewed at wileyonlinelibrary.com]

The patient developed a fever and right back pain on POD 1. Blood testing showed persistent leukocytosis and elevated CRP. Therefore, contrast‐enhanced CT was performed on POD 5, and a subcapsular renal hematoma and grade 3 hydronephrosis were observed. There was no active bleeding; however, an infected hematoma was suspected (Fig. 3). Several residual stones formed a stone street in the mid‐ureter, obstructing the stent and resulting in hydronephrosis. CT‐guided percutaneous drainage was performed on POD 9 because the infection persisted despite antibiotic treatment. After drainage, the patient's fever, back pain, and leukocytosis and elevated CRP improved rapidly, but not completely. Therefore, percutaneous nephrostomy was performed on POD 12. Thereafter, his fever disappeared, and blood testing showed normal. He was discharged on POD 19. During the postoperative course, the patient had never developed coronary heart disease. One month later, all residual stones were removed by ECIRS. All these procedures were performed with cessation of the antiplatelets.

## Discussion

Today, URS becomes a safe and effective method owing to the advances in devices. Common complications of URS are infection, hematuria, ureteral perforation, ureteral stenosis, and mucosal damage. Overall incidence of complications after URS is 9–25%, and most complications are minor and do not require intervention.^2^ Subcapsular renal hematoma is a well‐known complication of ESWL but occurs rarely after URS. Several cases have been reported (Table 1). Bansal *et al*.^3^ reported the first case of subcapsular renal hematoma after URS in 2010, and the current incidence is 0.15–0.4%.^4^, ^5^, ^6^ The most common symptom is back pain, fever and dizziness owing to anemia. In cases of postoperative presentation of such symptoms, subcapsular renal hematoma should be considered as a differential diagnosis. Ultrasonography is the easiest way of diagnosis and should be performed first, followed by the confirmation with CT.

**Table 1:** Published reports of subcapsular renal hematoma after URS

	Number of patients	Age/sex	Stone location	Stone size (median)	Preoperative hydronephrosis	Operation tine (min)	Irrigation pressure	Presenting symptoms	Transfusion	Management
Bansal^3^	1	35/M	U1	10 mm	Moderate	–	–	Back pain Fever	–	Percutaneous nephrostomy Percutaneous drainage
Bai^6^	11	44/82%M	U1 21% U2 29% U3 50%	14 mm	Mild 8% Moderate 29% Severe 50%	32–50	239 cmH_2_0	Back pain Fever	7 patients	3 conservative
										6 percutaneous drainage
										2 open drainage
Chiu^5^	4	55/25%M	U1 100%	16 mm	Mild 25% Severe 75%	30–150	80 cmH_2_0	Back pain Fever	4 patients	1 conservative 1 angiogram 1 percutaneous drainage 1 open drainage
Xu^7^	1	31/F	R2	36 mm	No	140	–		1 patient	angiogram
Tao^4^	3	60/67%M	U1 33% U2 67%	12 mm	Severe 100%	40–90	–	Back pain Fever	1 patient	1 conservative 2 percutaneous drainage
Kozminski^8^	4	56/25%M	R2 100%	–	–	24–54	–	Back pain Fever, nausea	No	3 conservative 1 transferred
Zhang^9^	1	24/M	U1	15 mm	Moderate	–	–	Back pain	1 patient	Conservative
Resorlu^10^	1	48/M	U3	10 mm	Moderate	–	–	Back pain, fever	No	Percutaneous drainage
Paiva^11^	1	38/M	R2	8 mm	–	–	–	Back pain, fever, nausea	No	Conservative
Taken^12^	9	37/77%M	R2 33% U1 33% U2 22% U3 11%	12 mm	Mild 22% Moderate 56% Severe 22%	30–80	–	Back pain, fever	3 patients	8 conservative 1 percutaneous drainage
Nazer^13^	1	45/F	R2	20 mm	No	88	80 cmH_2_O	Fever	1 patient	Conservative
Rose^14^	1	32/–	R3	12 mm	–	30	80 cmH_2_O	Fever	No	Percutaneous nephrostomy Percutaneous drainage
Watanabe^15^	1	39/M	R3	5 mm	No	94	–	Back pain	No	Conservative

The mechanism by which subcapsular renal hematoma develops after URS has not been clarified. Wei *et al*.^4^ reported that underlying diseases, such as hypertension, diabetes, and urinary tract infections, are important factors. Bai *et al*.^6^ reported that stone size, degree of preoperative hydronephrosis, increased operation time, and higher perfusion pressure during water irrigation are closely related to the formation of subcapsular hematoma. In a recent report, the authors hypothesized that manipulating a guidewire or increased renal pelvic pressure during water irrigation can injure the pelvicalyceal system.^3^ A possible explanation is that increased renal pelvic pressure could cause an increase in renal venous pressure, which might induce subcapsular bleeding. Furthermore, high renal pelvic pressure could cause rupture of the renal pelvic fornix, which is the weakest area, anatomically, leading to hematoma formation with separation of the renal capsule and renal parenchyma.^3^

In our hospital, the perfusion pressure is usually set to 80–100 mmHg using a manually inflatable pressure bag, and the pressure may be temporarily increased to 150 mmHg in cases of poor visualization. To reduce the pressure in the renal pelvis, a ureteral access sheath is inserted when using a flexible ureteroscope. During the present surgery, there was only minimal mucosal damage or bleeding. The drainage from the access sheath was good. The surgery was completed in about 2 h. However, in cases of poor visualization due to sandy stone fragments, the perfusion pressure was temporarily increased. Therefore, this temporal increase in perfusion pressure might be one of the causes of subcapsular hematoma. Moreover, postoperative hydronephrosis caused by the stone street could more directly lead to an increased pressure in the renal pelvis. Furthermore, the patient had multiple risk factors such as hypertension, urinary tract infection, and antiplatelet drug use.

Conservative treatment is generally selected for a subcapsular renal hematoma. However, percutaneous drainage, vascular embolization, or open surgery can be performed, depending on the degree of bleeding, underlying diseases, and the presence of infection (Table 1).

## Conclusion

Subcapsular renal hematoma after URS is a rare but severe complication and should be considered if the patient complained back pain after URS.

## Author contributions

Narumi Harada: Writing – original draft. Junji Yatsuda: Writing – review and editing. Ryoma Kurahashi: Writing – review and editing. Yumi Fukushima: Writing – review and editing. Takuya Segawa: Writing – review and editing. Takanobu Motoshima: Writing – review and editing. Yoji Murakami: Writing – review and editing. Takahiro Yamaguchi: Writing – review and editing. Yutaka Sugiyama: Writing – review and editing. Tomomi Kamba: Writing – review and editing.

## Conflict of interest

The authors declare no conflict of interest.

## Approval of the research protocol by an Institutional Reviewer Board

This case report was approved by Ethics Committee for Epidemiological and General Research at the Faculty of Life Science, Kumamoto University (Ethics No.1902).

## Informed consent

A written informed consent was obtained from the patient.

## Registry and the Registration No. of the study/trial

N/A.
